# Buried penis; what buried the penis?

**DOI:** 10.3389/fped.2025.1590147

**Published:** 2025-06-02

**Authors:** Mohamed Fawzy, Ahmed T. Hadidi

**Affiliations:** Hypospadias Centre, Sana Klinikum Offenbach, Offenbach am Main, Germany

**Keywords:** buried penis, hypospadias, fascia, smooth muscle fibers, collagen

## Abstract

**Objectives:**

To investigate the histological and immuno-histochemical features of dartos fascia in buried penis (BP) as compared to dartos fascia in hypospadias and normal children.

**Materials and methods:**

The study included 40 children, operated on in our center between January 2023 and January 2024. Patients were divided into 3 groups; **group A**: 13 patients with BP, **group B**: 14 patients with different grades of distal Hypospadias, and **group C** with 13 patients who were referred for circumcision (control group). All dartos fascia specimens were blindly examined by the same pathologist. The 3 groups were assessed for histological findings including collagen, elastin, nerve fibers, tactile bodies, fat, smooth muscles.

**Results:**

In group A (BP), there was statistically significant dominance of thick collagen fibers (thick fibers) *p* < 0.001, thick smooth muscle fibers (*P* < 0.001), thick convoluted nerve fibers (*p* = 0.004) and less fat (*P* < 0.001) as compared to the hypospadias and control groups. In the hypospadias group, intermediate collagen fibers were the predominant type of fibers (*p* < 0.001), in addition to long, thin and short thin elastin fibers (*p* < 0.001) compared to the buried penis and the control groups. The hypospadias group also had significant predominance of chaotic disorganized nonparallel smooth muscle fibers *p* = 0.003.

**Conclusion:**

The fascia in BP is characterized by abnormally thick collagen fibers, thick smooth muscle fibers and thick convoluted nerve fibers. This may explain why the penis is drawn inwards in BP and suggests that it is probably recommended to excise this abnormal fascia during the surgical correction.

## Introduction

Buried Penis (BP) is an uncommon anomaly first described by Keyes in 1919 as “an apparent absence of the penis which exists when the penis lacks its proper sheath of skin, lies buried beneath the integument of the abdomen, thigh or scrotum” (1). Since then, the term has been randomly used to include buried penis (2), concealed penis (3), inconspicuous penis (4), hidden penis (5), congenital mega-prepuce (6), trapped penis (7) and webbed penis (5).

It is time to avoid confusing terms in literature: Buried Penis (BP) may be defined as “an apparent absence of the penis characterized by an abnormally long inner prepuce (LIP). It is a rare congenital anomaly (since birth) with a wide spectrum of presentation. Concealed (hidden or inconspicuous) Penis (CP): should be reserved for acquired conditions presenting later in life due to abnormal excess fat accumulation in the genital area (8).

There are more than 200 publications dealing with BP in literature, however, we could only identify 4 studies dealing with the etiology and histology of dartos fascia in buried penis (9–12) rendering it an understudied topic.

We aim to examine the histological and immuno-histochemical features of dartos fascia in BP as compared to dartos fascia in hypospadias and normal children.

## Patients and methods

### Patients and groups allocation

The study included 40 children, operated on in our center between January 2023 and January 2024. Patients were classified into 3 groups; **Group A**: 13 patients with BP, **Group B**:14 patients with different grades of distal hypospadias, and **Group C** with13 children who were referred for circumcision for non-medical reasons (control group).

Ethical approval as well as informed written consents were obtained in all the patients.

Only children with congenital buried penis (since birth) were included in Group A. Children where the penis was concealed due to obesity, excess suprapubic fat or trapped penis or pure Mega-meatus were excluded. Children that underwent previous surgery or circumcision prior to correction of buried penis were also excluded.

**Figure 1:**
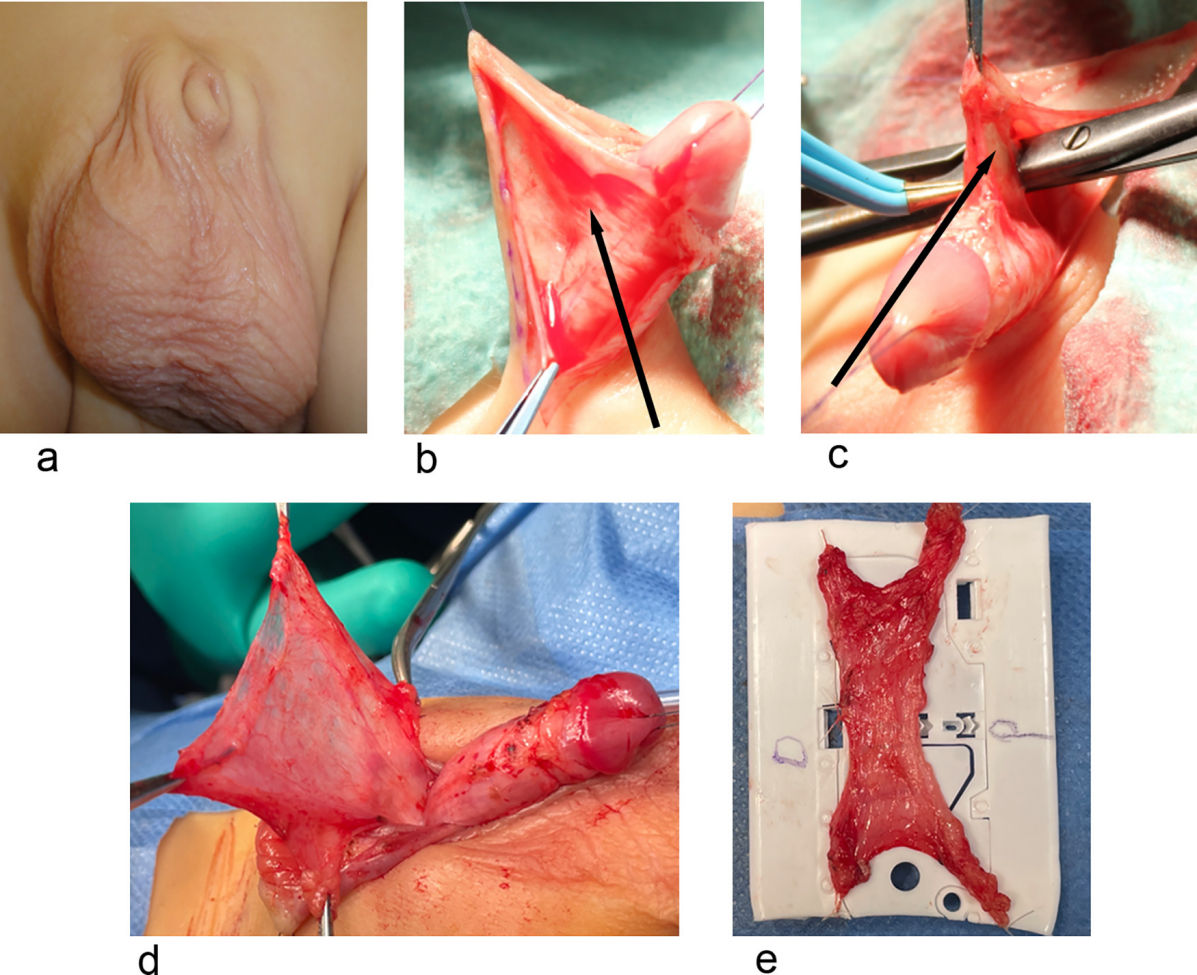
Group A. **(a)** A patient with BP. **(b,c)** The abnormal attachment of dartos fascia can be clearly seen in. **(d,e)** Show dissection of the complete dartos fascia and fixation on a plastic sheet and markation with a suture for orientation.

In group A (BP), Correction of buried penis included degloving, excision of the dartos fascia and fixation of the base of the penis to the pubis with non-absorbable suture as well as circumcision. Although the whole dartos fascia was excised, dartos fascia biopsies (0.5 cm in size) were specifically examined between 2 and 4 o'clock (Figure 1).

**Figure 2:**
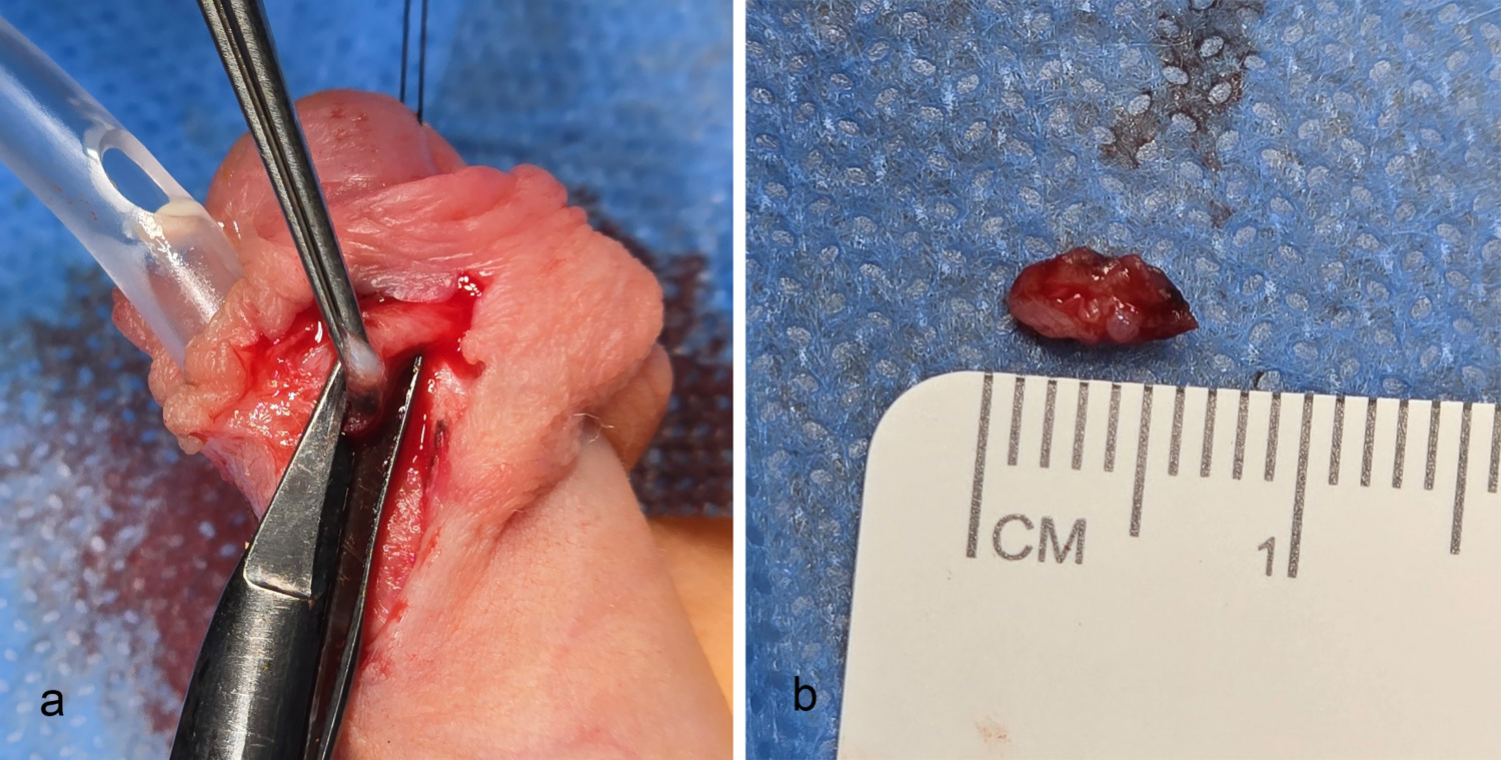
Group B. Patient with distal hypospadias. In hypospadias, the prepuce is deficient at 6 o’clock. **(a)** The dartos fascia was taken between 2 and 4 o'clock. **(b)** The biopsy was 0.5 cm in size.

In group B, children with proximal hypospadias or previous hypospadias repair were excluded. The biopsy was taken between 2 and 4 o'clock in patients with distal hypospadias before degloving (Figure 2).

In group C (control group), only children who underwent circumcision for cultural or religious were included. Children with conditions like lichen sclerosis, phimosis or history of balanitis were excluded. The biopsies were also taken between 2 and 4 o'clock.

### Preparation of the specimen

All the dartos fascia biopsies in the 3 Groups were carefully examined between 2 and 4 o'clock (Figures 1, 2). The biopsies were immersed in 4% buffered formalin. They were later embedded in paraffin and horizontal 4 μm sections were prepared.

### Histological and immune histochemical examination

Histological examination was carried out using hematoxylin-eosin (H/E) and Elastic van Gieson (EvG) stain to evaluate the presence and distribution of elastic fibers. The histochemical stains Factor 8 antibodies, Smooth muscle Actin (SMA) and S100 antibodies for nerve fibers (Dako, Glostrup, Denmark) were used to evaluate blood vessel, smooth muscle and nerve fibers distribution and thickness. Sections were stained using a Dako autostainer with the Dako EnVision FLEX þ detection system (Dako, Glostrup, Denmark). The system detects primary mouse and rabbit antibodies, and the reaction was visualized by EnVision FLEX DAB þ Chromogen. Using EnVision FLEX þ Mouse (LINKER) or EnVision FLEX þ Rabbit (LINKER) (Code K8019), signal amplification of primary antibodies was achieved. Deparaffinization, rehydration and heat-induced epitope retrieval (HIER) were carried out in one step with the three-in-one procedure buffer (Dako, Glostrup, Denmark, Target Retrieval Solution), pH 9 high [(10) (3-in-1) Code S2375]) at 97 C using a PT Link, Pre-Treatment Module 6 (Dako). Tissue samples were analyzed by light microscopy after 8 min counterstaining with Meyer's hematoxylin (Dako).

All sections were examined blindly by the same pathologist (CP) (from another university who was unaware of the diagnosis and surgical techniques used in our institution). She compared the findings of the different groups; Examination was done within less than one week of collection of the specimen to avoid any artifact caused by formaldehyde. The 3 groups were assessed for different histological and immunohistochemical criteria including collagen, elastin, nerve fibers, fat, smooth muscles and tactile bodies.

The collagen bundles were considered thin when less than 50 Micrometer, intermediate when between 50 and 100 Micrometer and thick when more than 100 Micrometer.

The nerves were considered thin when (<60 Micrometer), Intermediate (60–90 Micrometer) and thick (>90 micrometer).

### Statistical analysis

Statistical analysis was done by IBM SPSS for Windows, version 27 (IBM Co., Armonk, NY, USA). Numerical data were presented as the median and interquartile range (IQR), analyzed by Kruskal–Wallis test. Categorical data were presented as the frequency and percentage analyzed by Chi-square test. A two tailed *P* value < 0.05 was considered statistically significant.

## Results

Forty patients were included in this study; All children in the 3 groups were less than 4 years old. The median age was 19.5 months with IQR ranges between 15 and 42 months. The 3 groups were assessed for different histological criteria including collagen, elastin, smooth muscles, nerve fibers, fat and tactile bodies. Intra-operatively, it was observed that the dartos fascia in patients with BP had thick abnormal attachment to the body of the penis below the coronal sulcus (personal observation) (Figures 1b,c) (Supplementary Tables S1–S3).

The measurement of the **nerve bundles** in Group A was 90–110 Micrometer, in Group B was 70–100 Micrometer and Group C was 35–60 Micrometer.

The measurement of the **collagen bundles** in Group A was 70–250 Micrometer, in Group B was 50–100 Micrometer and in Group C was 20–70 Micrometer (Supplementary Table S1).

Spinoit classification of **smooth muscle fibers** (mentioned in detail in the discussion) was used in this study (Supplementary Table S2) (9).

**A new examination in the current study** is the examination of fat, nerve fibers and Vater tactile bodies content in the 3 groups (Supplementary Table S3).

**To summarize the findings of the study:**

**Figure 3:**
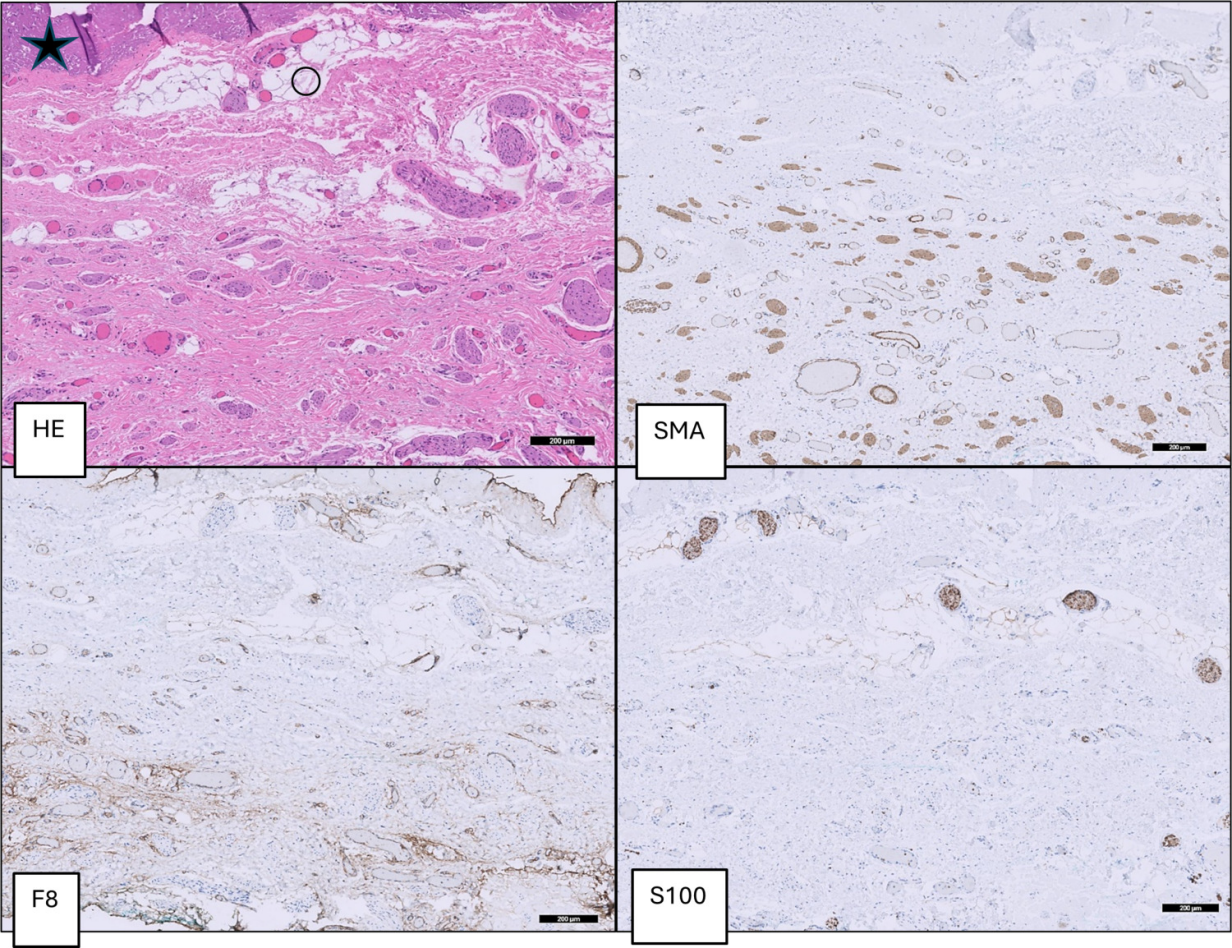
Group A (BP): dartos fascia of a patient with buried penis with thick parallel oriented muscle fibers (SMA), thick collagen fibers (*) and large blood vessels (F8) and thick convoluted nerve fibers (S100).

**Figure 4:**
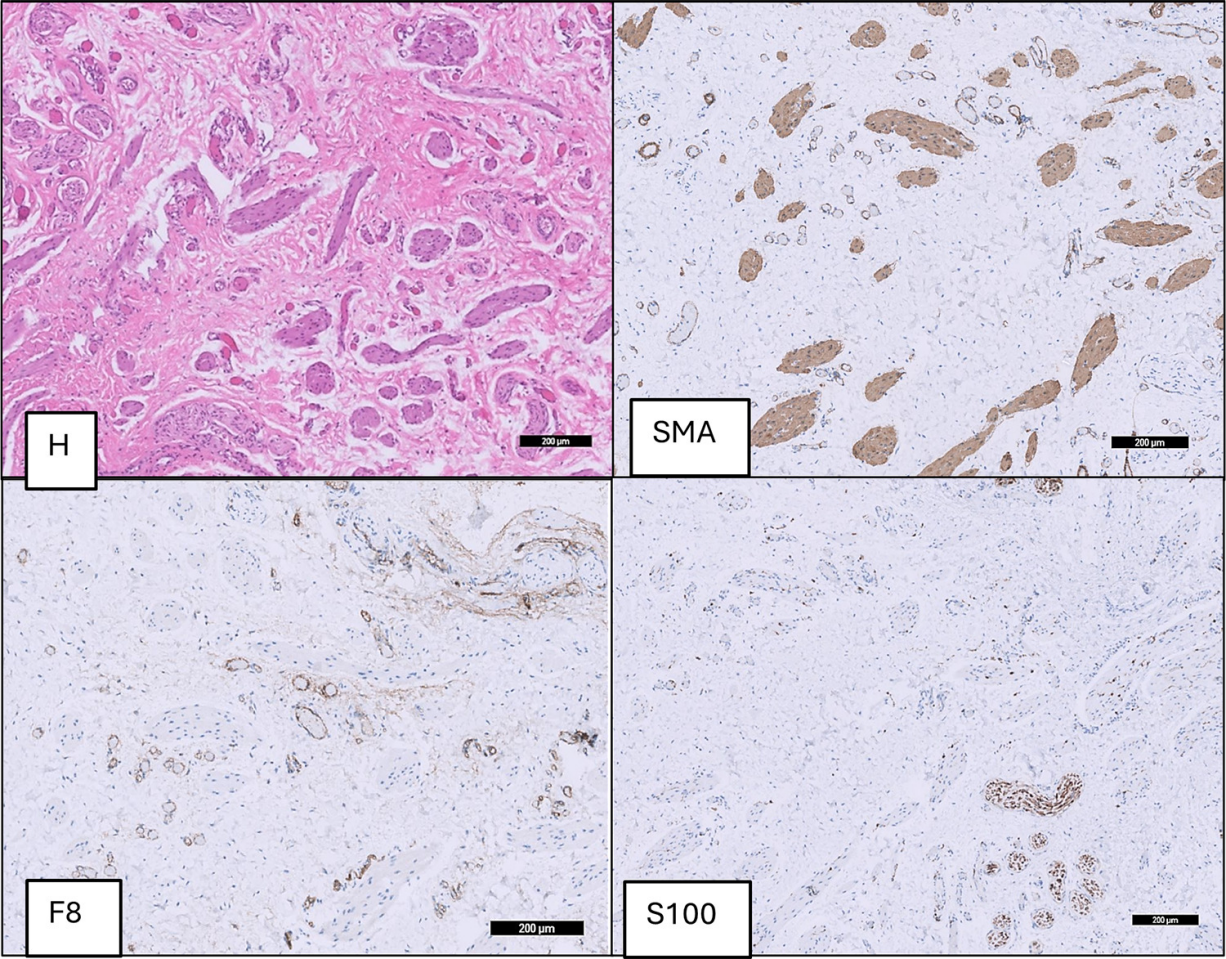
Group B (hypospadias): dartos fascia of a hypospadias patient with thicker chaotic, disorganized non parallel muscle fibers (SMA), intermediate collagen fibers, short thin elastin fibers and alternating size of blood vessels (F8) and nerve bundles (S100).

**Figure 5:**
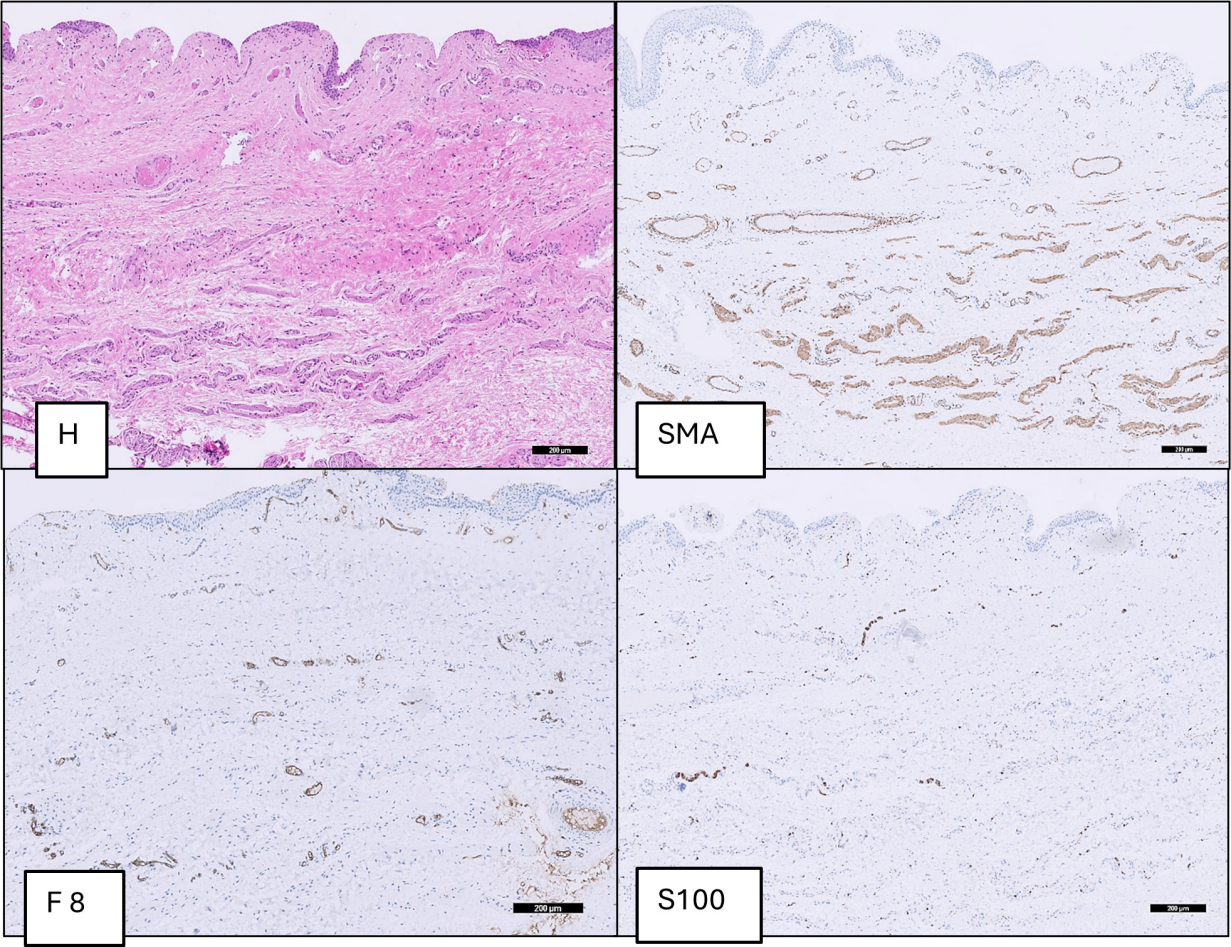
Group C (control group): dartos fascia of a circumcised patient with small parallel oriented muscle fibers (SMA), thin collagenous fibers and mostly small size of blood vessels (F8) and nervous bundles (S100).

**The Buried Penis (group A),** (Figure 3), was characterized by statistically significant dom- inance of thick collagen fibers (thick fibers) *p* < 0.001, thick smooth muscle fibers (*P* < 0.001) and thick and convoluted nerve fibers (*p* = 0.004) as compared to hypospadias (B) and control (C) groups. There was no chaotic disorganized smooth muscle fibers.
**In the hypospadias group (group B),** (Figure 4) intermediate collagen fibers were the pre- dominant fibers (*p* < 0.001), in addition to thin elastin fibers (*p* < 0.001) compared to the buried penis (A) and the control (C) groups (Figure 5).
**The hypospadias group (B)** also had significant predominance of *chaotic disorganized, nonparallel* smooth muscle fibers *p* = 0.003, while the smooth muscle fibers were significantly thicker in the buried penis group (A) *P* < 0.001. This was not seen in group A or C.
**The control group (C)** had organized parallel smooth muscle fibers (61.5%), long thick elastin fibers (100%), thin collagen fibers (76.9%) and no chaotic disorganized smooth muscle fibers.
**An additional new examination** in the current study is the examination of fat, nerve fibers and Vater tactile bodies content in the 3 groups (Supplementary Table S3). ***The buried penis fascia had statistically significant very low fat tissue (only 1 in 13 patients) as compared to the presence of fat tissue in all the control group patients (p*** ***<*** ***0.001)*.**

In addition, the buried penis fascia contained statistically significant thicker and more convoluted nerve fibers (Supplementary Table S3) compared to the hypospadias and control groups (*p* < **0.004).**

Although Vater-Pacini-tactile bodies were predominant in the buried penis group there was no statistically significant difference compared to the other two groups.

## Discussion

Fascia covers every structure of the body, creating a structural continuity that gives form and function to every tissue and organ (13). Superficial fascia describes the membranous layers that lie directly under the skin with loosely packed interwoven collagen and elastic fibers to maintain the extracellular matrix (14).

Dartos fascia (superficial fascia) of the penis is an example of muscle containing superficial fascia. These smooth muscle bundles extend and attach to the layer above (skin) and the layer below (Bucks fascia). Dartos fascia provides the blood supply to the skin and allows degloving without ischemia of penile skin (15, 16). Proximally a thin layer deeper to the dartos fascia called “*tela superficialis*” is covering the extracorporeal parts of cavernous vessels and nerves (17–19).

The exact etiology of buried penis remains unclear. However, inelasticity of dartos fascia (2, 20), excess prepubic fat and abnormally loose attachment of skin and superficial fascia to deep fascia (16, 21) are among the proposed theories to explain the etiology of buried penis. Buried penis is also characterized by an abnormally long inner prepuce (LIP) (8).

Buried penis can lead to psychological disorders, problems with voiding and recurrent balanitis if left uncorrected (9, 22). Only very few studies in literature examined the histology of dartos fascia in buried penis (9–12).

Spinoit et al. study included 18 patients with buried penis and 94 patients with hypospadias and identified 3 histological patterns. **Pattern I** (normal) included smooth muscle fibers of dartos fascia that had a parallel configuration in the subcutaneous tissue**. In pattern II** smooth muscle fibers were underdeveloped and hypo- trophic, while in **pattern III** the smooth muscle fibers were not parallel and randomly distributed (9).

Atmoko et al. studied 20 patients with buried penis, in whom, dartos fascia was completely excised after complete degloving. The study concluded that the dartos fascia in buried penis and hypospadias are abnormal and contain a smaller number, however, thicker collagen and elastin fibers compared to the normal control group in addition to increased reticulin (Type III collagen) to total collagen ratio (10).

Our study confirmed that and showed that buried penis group (A) had significantly thicker collagen fibers compared to the hypospadias group (B) and control group (C).

Zhang et al. excised the dartos fascia in 49 older children with buried penis and compared the histology with the fascia from 20 men cadavers. They concluded that excision of the dartos fascia resulted in longer penises. They also observed that the dartos fascia in buried penis contained disordered fragmented elastin (11). In the present study both elastin and collagen fibers were significantly thick in the buried penis group as compared to the control and hypospadias groups.

The Spinoit classification was used in the current study (9). Our results showed that the buried penis group had predominantly thick collagen fibers (92.3%), thick long elastin fibers (76.9%) and **no** thin short elastin fibers and **no** thin collagen fibers.

The buried penis group had abundant thick and intermediate smooth muscle fibers that were characteristically parallel. On the other hand, 42% of the hypospadias group was characterized by irregular chaotic non parallel smooth muscle fibers.

Kurtuluş et al. concluded that penile retraction is due to an increased contractility in the smooth muscles, caused by over expression of SM 1 of the smooth muscles myosin heavy chain isoforms of the dartos fascia, leading to elevated SM2/SM1 ratio (12).

To out knowledge, **this is the first study** to examine fat, nerve fibers and Vater tactile bodies content in the 3 groups. The study showed statistically significant low fat tissue (only 1 in 13 patients) (*p* < 0.001). This finding contradicts the postulation that congenital buried penis is due to excess fat (16, 20).

In addition, the buried penis fascia contained statistically significant thicker and more convoluted nerve fibers compared to the hypospadias and control groups (*p* < **0.004)**, suggesting abnormal innervation of the fascia in the buried penis.

One question that may rise is, whether the preputial dartos fascia is similar in structure to the penile dartos fascia. Embryologically, the preputial dartos fascia develops from the penile dartos fascia (23). Anatomically, the preputial dartos fascia is in direct continuity with the penile dartos fascia. Histologically, there seems to be no histological difference between them (11, 17, 18, 24).

The findings of the study have important clinical implications. It was our practice to freely detach (without excision) the dartos fascia off the penile body to free the penis and avoid retraction (8). Now, we completely excise the abnormal dartos fascia in patients with buried penis (Figures 1,2). This has 2 advantages: It helps to close the penile skin as the edematous dartos fascia is excised and it keeps the penis fixed outside the body. This also confirms the findings of Atmoko et al. (10) and Zhang et al. (11).

**The strong points of the study** include that it is a prospective study, the pathologist was blinded regarding the pathology of the patient, and the special care given in preparation and examination of the specimens to minimize bias and the additional examination of fat, nerve fibers and tactile bodies.

One important concern is whether excision of the dartos fascia may cause ischemia and necrosis of the penile skin. There is a collateral arterial supply from the superficial and deep external pudendal branches of the femoral arteries as well as the superficial vessels of the groin and perineum that branch from the internal pudendal and deep inferior epigastric arteries. These varied arteries contribute to an anastomotic circle of vessels at the base of the penis. Even if the dartos fascia is surgically mobilized, the vascular plexus of subdermal vessels is able to sustain the penile skin (25). Surgical popular examples are the Duckett island tube and the Onlay island flap that are based on dartos fascial flaps that are mobilized ventrally for urethroplasty without jeopardizing the penile skin.

**There are several limitations in this study**, this includes the small sample size [buried penis is an uncommon anomaly (8)], and the short term follow up. However, this study focuses on histological and immuno-histochemical findings rather than the surgical technique and outcome. Another possible limitation is probably that biopsies were taken from similar sites in the 3 groups. However, the study was specifically designed to compare the dartos fascia from the exact location in the 3 groups. It is worth mentioning that examination of the whole dartos fascia in BP showed homogenicity and there was no difference between biopsies taken at 2 o'clock or dorsally at 12 o'clock.

## Conclusion

The fascia in BP is characterized by abnormally thick collagen fibers, thick smooth muscle fibers and thick convoluted nerve fibers and is less elastic. This may help to explain why the penis is drawn inwards in BP and suggests that excision of this abnormal dartos fascia, rather than detachment during the surgical correction, may help to reduce recurrence.

## Data Availability

The original contributions presented in the study are included in the article/Supplementary Material, further inquiries can be directed to the corresponding author.
