# Supplementary material for: Buried penis; what buried the penis?
Source: Front Pediatr. 2025 Jun 2;13:1590147. doi: 10.3389/fped.2025.1590147 (PMC12171150; doi:10.3389/fped.2025.1590147)
Supplement: Supplementary file 1 [file Table1.docx]

**Supplemtary table 1:** Comparison of Elastin and Collagen in the three groups

|  |  | **Group A (N=13)** | **Group B (N=14)** | **Group C (N=13)** | **P-value** |
| --- | --- | --- | --- | --- | --- |
|  |  | **Buried penis** | **Hypospadias** | **Control** |  |
| **Elastin** | Short thin fiber fragments | 0 (0%) | 3 (21.4%) | 0 (0%) | **<0.001*** |
|  | Thin long fibers | 3 (23.1%) | 7 (50%) | 0 (0%) |  |
|  | Thick long fibers | 10 (76.9%) | 4 (28.6%) | 13 (100%) |  |
| **Collagen** | Thin fibers | 0 (0%) | 6 (42.9%) | 10 (76.9%) | **<0.001*** |
|  | Intermediate thick fibers | 1 (7.7%) | 8 (57.1%) | 3 (23.1%) |  |
|  | Thick fibers | 12 (92.3%) | 0 (0%) | 0 (0%) |  |
