# Supplementary material for: Buried penis; what buried the penis?
Source: Front Pediatr. 2025 Jun 2;13:1590147. doi: 10.3389/fped.2025.1590147 (PMC12171150; doi:10.3389/fped.2025.1590147)
Supplement: Supplementary file 2 [file Table2.docx]

**Supplementary Table 2**: Comparison of smooth muscle fibers in the three groups

|  |  | **Group A (N=13)** | **Group B (N=14)** | **Group C (N=13)** | **P-value** |
| --- | --- | --- | --- | --- | --- |
|  |  | **Buried penis** | **Hypospadias** | **Control** |  |
| **Spinoit** | Organized parallel fibers | 6 (46.2%) | 2 (14.3%) | 8 (61.5%) | **0.003*** |
|  | Hypotrophic not well developed fibers | 7 (53.8%) | 6 (42.9%) | 5 (38.5%) |  |
|  | Chaotic disorganized fibers not parallel | 0 (0%) | 6 (42.9%) | 0 (0%) |  |
| **Thickness** | Thin fibers | 0 (0%) | 1 (7.1%) | 9 (69.2%) | **<0.001*** |
|  | Medium thickness | 2 (15.4%) | 9 (64.3%) | 4 (30.8%) |  |
|  | Thick fibers | 11 (84.6%) | 4 (28.6%) | 0 (0%) |  |
