# Supplementary material for: Buried penis; what buried the penis?
Source: Front Pediatr. 2025 Jun 2;13:1590147. doi: 10.3389/fped.2025.1590147 (PMC12171150; doi:10.3389/fped.2025.1590147)
Supplement: Supplementary file 3 [file Table3.docx]

**Supplementary Table 3**: Comparison of fat, nerve fibers and tactile bodies in the three groups

|  |  | **Group A (N=13)** | **Group B (N=14)** | **Group C (N=13)** | **P-value** |
| --- | --- | --- | --- | --- | --- |
|  |  | **Buried penis** | **Hypospadias** | **Control** |  |
| **Fat tissue** | Fat tissue existing | 1 (7.7%) | 11 (78.6%) | 13 (100%) | **<0.001*** |
|  | Fat tissue not existing | 12 (92.3%) | 3 (21.4%) | 0 (0%) |  |
| **Tactile bodies** | Vater pacini tactile bodies present | 7 (53.8%) | 12 (85.7%) | 10 (76.9%) | 0.200 |
|  | No tactile bodies present | 6 (46.2%) | 2 (14.3%) | 3 (23.1%) |  |
| **Nerve fibers** | Single thin nerves | 0 (0%) | 6 (42.9%) | 3 (23.1%) | **0.004*** |
|  | Intermediate number of fibers | 9 (69.2%) | 8 (57.1%) | 10 (76.9%) |  |
|  | Thick nerves with convolution | 4 (30.8%) | 0 (0%) | 0 (0%) |  |
